# Utilization of Red Mud as a Source for Metal Ions—A Review

**DOI:** 10.3390/ma14092211

**Published:** 2021-04-25

**Authors:** Sneha Samal

**Affiliations:** FZU-Institute of Physics of Czech Academy of Sciences, Na Slovance 1999/2, 182 21 Prague, Czech Republic; samal@fzu.cz; Tel.: +42-266-05-2361

**Keywords:** red mud, resources, fly ash, reduced slag, metal ion recovery, mineralogical

## Abstract

An overview is presented on the prospective use of red mud as a resource in this review. Various scopes are suggested for the utilization of red mud to maintain a sustainable environment. The potential use of red mud covers the valuable metal recovery that could emphasize the use of red mud as a resource. Red mud could act as reduced slag in the metallurgical field for the extraction of minerals and metals for upscale application. Although many studies have revealed the potential utilization of red mud, most of them are only limited to a lab-scale basis. Therefore, a large-scale investigation on recycling of red mud for the extraction in the area of the metal recovery section will draw attention to the extensive use of red mud. Metal ions of major elements Fe (44 wt.%), Al (18.2 wt.%), Si (14.3 wt.%), Ti (9.3 wt.%), Na (6.2 wt.%), Ca (4.4 wt.%) as major elements and of Mg, V, Mn, Cr, K as minor elements and rare earth elements such as Ce (102 mg/kg), La (56 mg/kg), Sc (47 mg/kg), Nd (45 mg/kg), Sm (9 mg/kg). Moreover, an appropriate in-house metal recovery facility with the alumina industry will come out as a cost–benefit analysis.

## 1. Introduction

Red mud is one of the by-products generated in the aluminum industry from the ore of bauxite during the calcination process for the extraction of aluminum dioxide. The term “red mud” is established and derived from the two words of “red”, which refers to the color, and “mud”, which refers to the waste generated after the alumina extraction from the bauxite ore, by a calcination process. Generally, 2.5–3 kg of red mud is produced in each 1 kg of Al production from the bauxite industry [1]. As the global production of aluminum is approximately 64 million tons, this result in 160 million tons of red mud to dispose of. The current method of red mud disposal is to simply pump it into ponds or dry up the red mud with a special liner [2]. In both approaches, a large amount of land is used and ultimately the land should be maintained properly, rather than disposing of the product as waste to the surrounding area, causing serious environmental issues and health hazards. The alkaline nature of red mud and dried-up dust disposable to the environment could be minimized by spraying water on the dry red mud powders. Furthermore, the alkaline nature of red mud inhibits the vegetation growth in those areas, thus it must be corrected by adding acidic flux before its disposal into the surroundings. Given all these environmental implications, it would be appropriate to think of a new use for red mud. “Waste is a resource if we use it. Otherwise, it is waste if we waste it” [3]. Thus, the red mud residue, after the extraction of the minerals, could be considered as a potential building material for the construction of roads, landfill sites, and building materials. Recently, a combination of red mud–fly ash composite could find application in the preparation of geopolymers as an alternative material for the construction industry [4,5].

The recovery of critical raw materials from red mud involves many benefits including environmental, social, financial, economic, and technological benefits [6,7]. The content of metals such as Ti, Si, Fe, Na, and Al in red mud is 2–12%, 1–9%, 14–45%, 1–6% and 5–14% respectively. Apart from representing a huge solution in the construction sector, when present in a large quantity, red mud as a resource opens up various possibilities for the extraction of minerals and ions such as the major elements Fe, Ti, Mn, Al and Ca, Na, Si, Cr, Mn, V, La, Sc, Y. Rare earth elements (REE) such as Ce (102 mg/kg), La (56 mg/kg), Sc (47 mg/kg), Nd (45 mg/kg), Sm (9 mg/kg) are also valuable elements present inside red mud. REE are the most important critical raw materials for the European Union [8]. Red mud can be also considered as sintered ceramics for electroceramic materials [9,10,11].

In powder technology, red mud could be considered as resource for the recovery of metals such as Fe, Ti, Mn, Na, K [12,13]. Simultaneously, red mud could be used as a coating material for various composites against harsh environments and high-temperature sintering, against wear and corrosive behavior [14,15,16]. Unlike the recovery of metal ions, which will certainly not be the main business for the red mud, it could also act as the main component for construction and road fill materials [17,18]. Fly ash with acidic nature inhibits agglomeration of the volatilization of heavy metals at low temperatures within the red mud combination [19,20,21].

**Figure 1:**
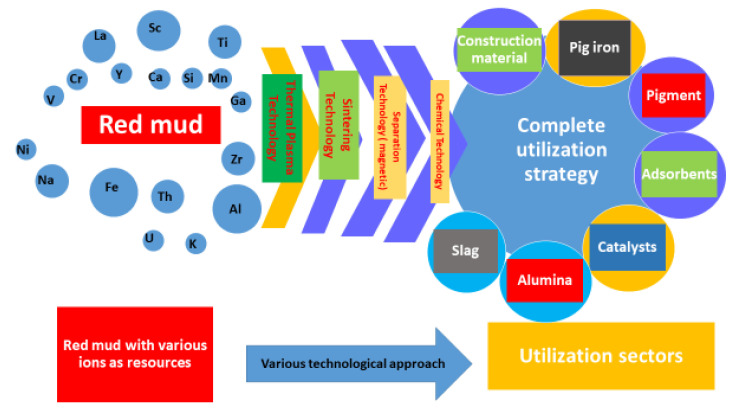
Scheme representing the technologies implemented in red mud for its complete utilization in various sectors.

In Figure 1 the combination of various technologies that could be implemented for a complete utilization strategy is shown. The comprehensive utilization of red mud as a resource opens up in various sectors such as red mud-based geopolymers in the construction and metal extraction industries. Synergetic utilization of red mud emerges as flue gas in the geopolymer industry sector for an alternative binder in cement material. The exploitation of these potential techniques, for metal extraction from red mud, is subordinate to the establishment of a small plant, near the aluminum industry, for resource utilization [22,23,24,25]. The reduction of red mud and fly ash mixtures proved the formation of reduced slag in the sintering process during lab-scale experiments. Based on the latter, it is possible to design a synergetic utilization for the red mud/fly ash mixture. It has been seen that the hazardous heavy metals could be recovered as alloy from the reduced slag [26,27]. Therefore, this environmentally friendly co-reduction process could be implemented as one sound solution for red mud and fly ash, leading to complete utilization of the resources, thus representing a zero-waste technology [28]. The optimized parameters for the reduction process were chosen as 20 wt.% fly ash with 80 wt.% red mud, at a temperature of 1100 °C for 2 h. The sintered slag contained CaO, SiO_2_, Al_2_O_3_, and FeO, as well as a glass phase, which is similar to ground-granulated blast-furnace slag and supports broad future applications. It has also been seen that the treatment of the red mud’s alkaline nature with an additive for surface modification will enhance the utilization on an upscale basis [29,30]. The major and minor elements of red mud are quantified in Table 1.

The composition shows the presence of heavy irons and minerals of Fe, Si, and Ti in the major quantity. Red mud could be considered as bricks, road surface material, and in the cement industry with potential use for building applications. However, this approach is limited due to the alkaline nature of the material. The alkaline nature of red mud is reduced by the acidic counterpart of fly ash, generating the neutral nature of the composite. The latter could be the most significant solution for the vastness of the problem, but careful consideration is required for this application. After the Al content, Fe represents the second-largest amount of metal that is separated by a magnetic separator. The nonmagnetic part of the residue can be considered as a construction material. Furthermore, some researchers searched for producing steel and cement from red mud [43]. Additionally, the recovery of Al, caustic soda, and lime could be used as catalyst for enhancing the Bayer process for increased Al production. However, despite the invaluable outcomes obtained from all techniques associated with red mud utilization, they are not practically suitable to use for recycling large amounts of red mud (currently 160 million tons annually [44]). The amount or iron in red mud is the largest, which, when disposed with red mud annually, represents a waste of metal [45]. Thus, metal recovery from red mud opens a wide field for potential utilization as resource.

The main aim of this review article is to present an up-to-date scenario in the area of red mud to be considered as a source of various metals, minerals, and rare earth elements. This review presents an overview of the status of red mud as a metal resource considered from the last 11 years. This article will hopefully encourage researchers, industralists, scientists, and government sector to use red mud as a source of metal ions.

## 2. Sources and Utilization of Red Mud

### 2.1. Relevant Sources for Literature Review

**Figure 2:**
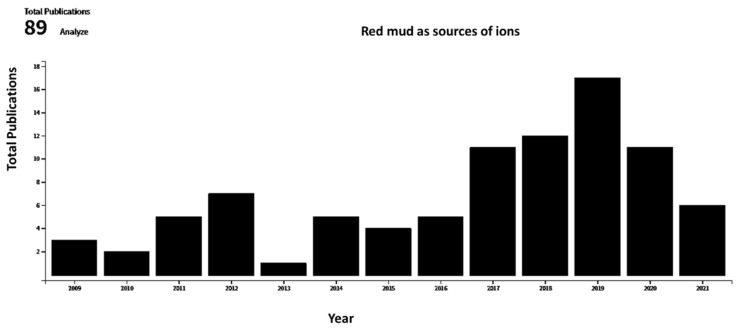
Total publications as function of year for red mud considered as source of ions (data collected from Web of Science).

A broad range of literature sources, dating from 1991 to 2021, in the areas of red mud and red mud composites were reviewed for this article. The databases searched for this literature survey include various sources such as MDPI, Scopus, Science Direct, Google Scholar, and Springer. Articles, conference proceedings, data, reviews, chapters, and books of similar topics were filtered using search terms such as “red mud”, “composite”, “mineral”, “microstructure evolution”, “metal ion recovery”, “mineralogical characteristics of the materials”. Section 1 in the introduction includes all the potential previous studies in this area. Section 2 includes various types of red mud and composites with potential applications. The basic and advanced application of red mud and composite is followed in Section 3 with emphasis on some recent literature surveys. Section 4 compares the data with the present scenario through an exhaustive literature survey. Figure 2 displays the total publication from 2010–2021 in the area of red mud that consider it as a source of metal and ions.

### 2.2. Utilization of Red Mud as Metal Resource

**Figure 3:**
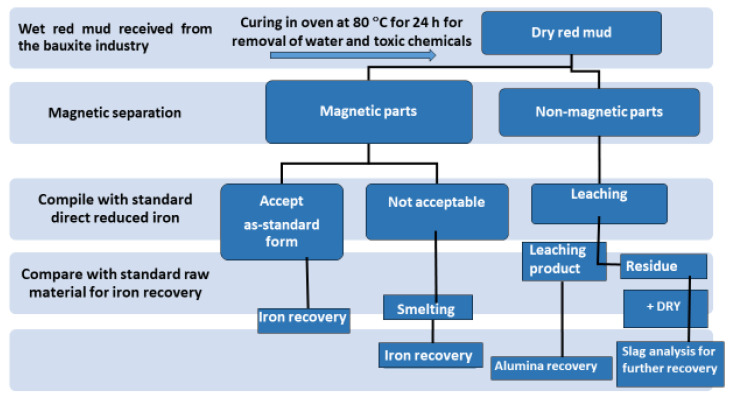
Schematic flow sheet on the iron, alumina, and slag for recovery of various metals.

Although researchers highlight that red mud is a large contributor to the construction sector, it is generally recognized as waste material. The term “waste” creates, both psychologically and from a media viewpoint, an obstacle in the application areas. Thus, replacing the term “waste” with “resource” could add significant interest in the extraction of minerals and their use. In this work, an investigation was carried out for review in the area of utilization of red mud as a source of metallic ions and resource material. Red mud, added with various weight percentages of fly ash to neutralize the acidity, undergoes the sintering process for the conversion into a reduced slag material. This sintered product could act as a basic resource for the extraction of metal ions and as a major by-product for the mineral industry. Figure 3 illustrates the red mud utilization from the as-received stage towards the final stage for industrial utilization.

**Figure 4:**
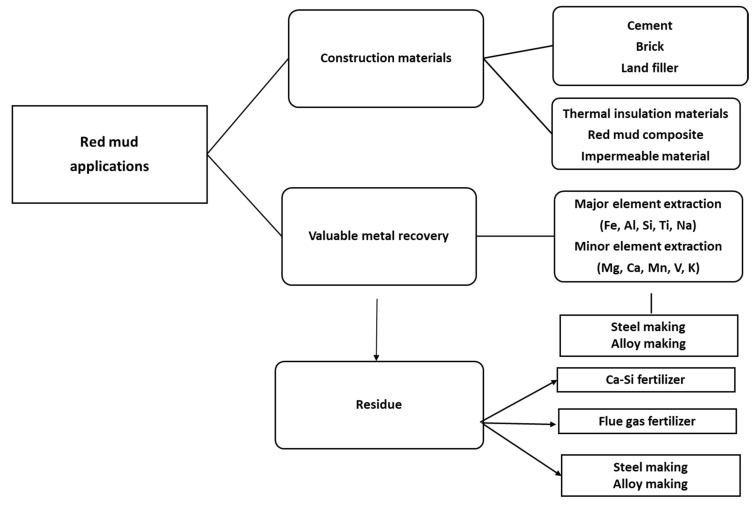
The various areas of red mud utilizations.

The dry red mud undergoes magnetic and non-magnetic separation that follows up the smelting process for iron recovery. Non-magnetic parts undergo the leaching process for Al recovery. Figure 3 portrays the flow sheet of various types of red mud utilization. Preliminary treatment involves the magnetic separations of bulk iron parts from the red mud. Accordingly, the magnetic and non-magnetic parts undergo different treatment in the further steps for iron recovery on the acceptable norms. If the magnetic parts are non-acceptable, they undergo smelting for iron recovery. The non-magnetic parts undergo leaching for alumina recovery and the residue undergoes slag recovery as the utilization of the major parts. A key point to benefit in terms of human resources and the economy could be the establishment of a plant for the beneficiation of red mud as resource alongside the bauxite industry. Particularly, to avoid transportation costs, the waste utilization facility processes and tools such as electric arc furnace, sintering of red mud, and leaching facility should be present in the proximal areas of the aluminum industry. One of the innovative processes in the production of pig iron is a by-product from reduced red mud by the carbothermal reduction process. The various process and active areas in which red mud can be treated can be divided into major and minor activities (Figure 4). Red mud can be used as a primary resource in the construction industry, for example, as bricks and other suitable materials for making houses, or as material for pavement. Red mud could be used in the industrial sector of iron recovery or metal extraction and smelting for the by-product of pig iron and calcium titanium-rich compounds for recovery of titanium. Finally, it can be used in the carbothermal reduction process for iron recovery, which could be a possible step for steel making. The rest of the residual red mud could be considered as the reductant for alumina recovery. Major use in the areas of construction and landfill opens the application of red mud in combination with metal recoveries such as Al, Fe, and its integrated combination towards the reduction process for the steelmaking. Integrating the red mud with other materials could improve its use in synergetic utilization [45].

Foaming ceramics are emerging as a new group of materials that could improve performance that could act as energy-saving materials [46]. Sintering and thermal plasma open the possibility of the synthesis of energy-saving materials by generating porosity in the sintered material [47,48]. In these cases, sintering is one of the effective processes of using carbo-thermal reduction inside the furnace that facilitates the formation of sintered slag. The quantity of fly ash content (wt.%) reduces the mixture of red mud and fly ash that undergoes chemical and physical reduction processes as a function of sintering temperature. The mineralogical evolution in the sintered product and the end-product was examined to confirm the presence of minerals and ions at the end of the process.

### 2.3. Sources of Metal Ions

In this article, an effort was made to create a review in the area of utilization of red mud as a source of metal ions. Various steps and process related to the mineralogical evolution of various metal and rare earth ions in red mud are covered and discussed. Simultaneously, the application of red mud in various fields is covered, where red mud could be given importance as a resource rather than waste.

Table 2 shows the red mud generated from various plants with different chemical compositions.

## 3. Physical and Chemical Properties of Red Mud

### 3.1. Particle Size Distribution and pH of Red Mud

**Figure 5:**
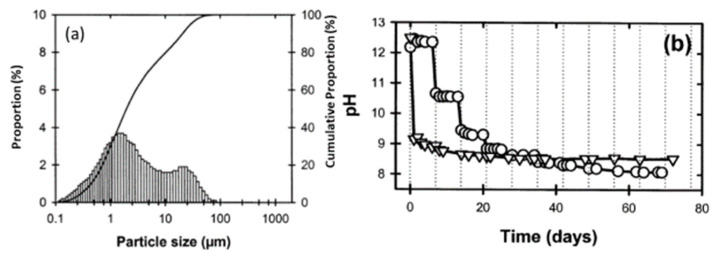
(**a**) Particle size distribution versus cumulative proportion in red mud as the residue from the alumina industry; (**b**) pH as a function of time. Reprint with permission from ref. [53]. Copyright 2004, Wiley.

Red mud, generated as waste in the aluminum industry and generally disposed of in the surrounding areas, was supplied from an Indian bauxite producer (Bharat Aluminium Company Ltd., BALCO, Korba, Chhattisgarh, India). Fly ash was supplied from the thermal power plant (Coal Plant, India). The slurry red mud received directly from the aluminum industry contains a lot of water and moisture. Red mud needs to be dried at 100 °C for 24 h, to remove the water and chemicals such as volatile compounds in a standard furnace in an air medium. The particle sizes of the red mud and pH change as a function of time, as shown in Figure 5 [53].

Red mud consists of various fine-size particles within the range of 0.1–100 µm. Average particles fall within the range of 1 µm. The alkaline nature of red mud decreases as the function of the day, from fresh red mud to aged, and becomes stable. Red mud was mixed with fly ash contents from 0 to 20 Vol% to observe the effect of neutralization of alkaline components with acidic flux by using agate mortar.

### 3.2. Ternary Phase Diagram of the CaO–Al_2_O_3_–SiO_2_ System

**Figure 6:**
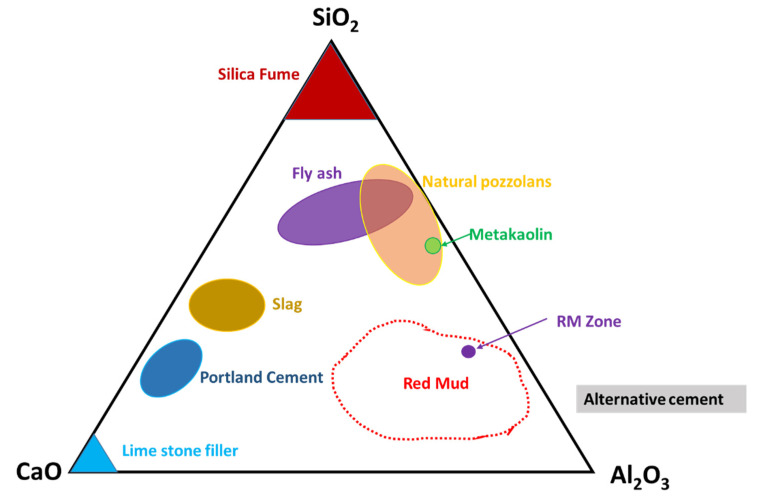
Ternary phase diagram of the CaO–SiO_2_–Al_2_O_3_ system that covers red mud and fly ash zones. Reprint with permission from ref. [54]. Copyright 2013, Elsevier.

Observation of the mineral compounds in the system of the CaO–Al_2_O_3_–SiO_2_ phase diagram reveals the red mud–fly ash falling into the category (Figure 6).

Thus, their presence in such a diagram could open up the possibility of mixtures of red mud and fly ash for utilization as an alternative cement category for construction purposes. The mixture falls within the zone of slag that could be boosted as a source of metal extraction as well and act as compatible material for alternative cement in the construction industry.

### 3.3. Phase Transformation during Thermal Decomposition

**Figure 7:**
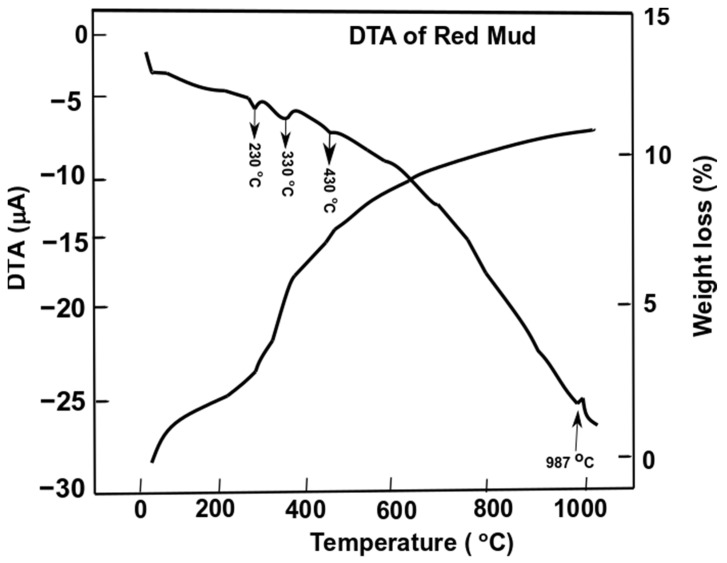
Differential thermal analysis of red mud with peak evolution and weight loss as a function of the temperature. Reprint with permission from ref. [55]. Copyright 2015, Elsevier.

Differential thermal analysis of red mud showed the combined effect of the decomposition reaction, concerning the weight loss and the associated energy changes. Figure 7 shows the evolution of red mud as the function of temperature concerning exo- and endothermic reactions [53].

Gibbsite phase emerges between 320 and 330 °C whichderives from the decomposition of x-Al_2_O_3_. The decomposition reaction of goethite FeO(OH) into hematite and water occurs as follows:

2α-FeO(OH) (s) → Fe_2_O_3_(s) + H_2_O (g) (1)

whilst the gibbsite decomposes into boehmite and x-alumina in the range of 230–330 °C

Al(OH)_3_ (s) → AlO(OH) (s) ↑(water evaporates) (2)

2AlO(OH) (s) → Al_2_O_3_(s) ↑ (water evaporates) (3)

Furthermore, goethite continues to decompose into hematite at 440 °C,

3 Fe_2_O_3_ (s) → 2Fe_3_O_4_ (s) + ½O_2_ (g) (4)

The alumina phase of red mud is very stable until higher temperatures. In the range of 900–1100 °C the formation of nepheline from cancrinite occurs

3CaO (s) + Al_2_O_3_ (s) → Ca_3_Al_2_O_6_ (s) (5)

and further decomposition reactions happen above 1100 °C

Fe_2_O_3_ (s) → 2FeO (s) + ½O_2_ (g) (6)

2FeO (s) + TiO_2_ (s) → Fe_2_TiO_4_ (s) (7)

3CaO (s) + Fe_2_O_3_ (s) +3 SiO_2_ (s) → Ca_3_Fe_2_Si_3_O_12_ (s) (8)

7Ca_3_Al_2_O_6_ (s) → Ca_12_Al_14_O_33_ (s) + 9CaO (s) (9)

Ca_3_Al_2_O_6_ (s) + Na_2_O (s) → Na_2x_Ca_3-x_Al_2_O_6_ (s) + xCaO (s) (10)

The weight loss of the sample of red mud is observed significantly towards higher temperatures. At 1000 °C, there is significant weight loss and more than 10 wt.% loss is observed. DTA analysis reveals the behavior of red mud sintered at a higher temperature.

### 3.4. Microstructure of Sintered Compound at 1100 °C Temperature

The sintering process further facilitates the mixture as the form of the pellet. Cylindrical pellets were prepared with a dimension of 0.5 × 2.5 cm^2^ by using water as a binder at a pressure of 50 MPa. The pellet of red mud–fly ash mixtures with various contents undergoes co-reduction in the graphite resistance furnace for sintering at various temperatures, 1000–1050–1100 °C, for a duration of 2 h in a static argon atmosphere, followed by cooling (2 h).

**Figure 8:**
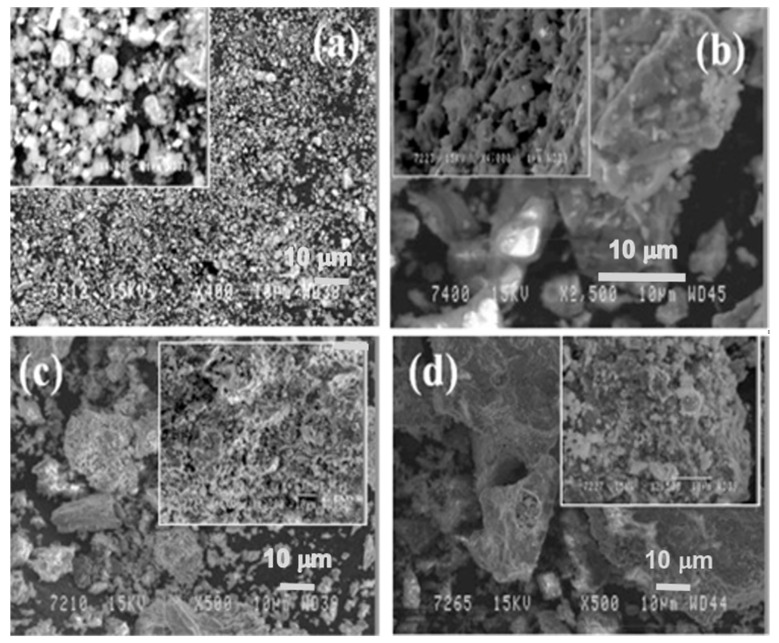
(**a**) Surface features of the red mud at RT; (**b**) sintered red mud without fly ash content; (**c**) sintered red mud with 10 wt.% of fly ash content; (**d**) sintered red mud with 20 wt.% of fly ash content at 1100 °C sintered temperature. Reprint with permission from ref. [55]. Copyright 2015, Elsevier.

In Figure 6, the microstructure evolution of as-received red mud in dry condition and sintered samples are presented. Globular particles, with fine size in a range from a few microns to the maximum particle size of 100 µm, are displayed (Figure 8a). The sintered composite of red mud-fly ash at the various wt.% shows the evolution of various phases as the function of temperature (Figure 8b–d). Iron phases of magnetite are shown in a lighter color and the darker region belongs to the quartz. Whereas, sintered red mud–fly ash composite shows isolated pores, elongated shape, and size of the crystals and ceramic matrix with some former phases. The sintered sample with 20 wt.% of fly ash shows that the porosity in sintered composite increased with irregular (Fe_3_O_4_), goethite (FeO(OH)), iron (Fe), hercynite (FeAl_2_O_4_), and aluminum silicates. Additionally, 20 wt.% of fly ash and sintering at various temperatures allows conversion of complex phases towards simpler phases of compounds of magnetite, iron, calcium aluminosilicate, sodium aluminum silicates, and Goethite phases [55,56].

### 3.5. Phase Evolution of Sintered Sample as a Function of Temperature

**Figure 9:**
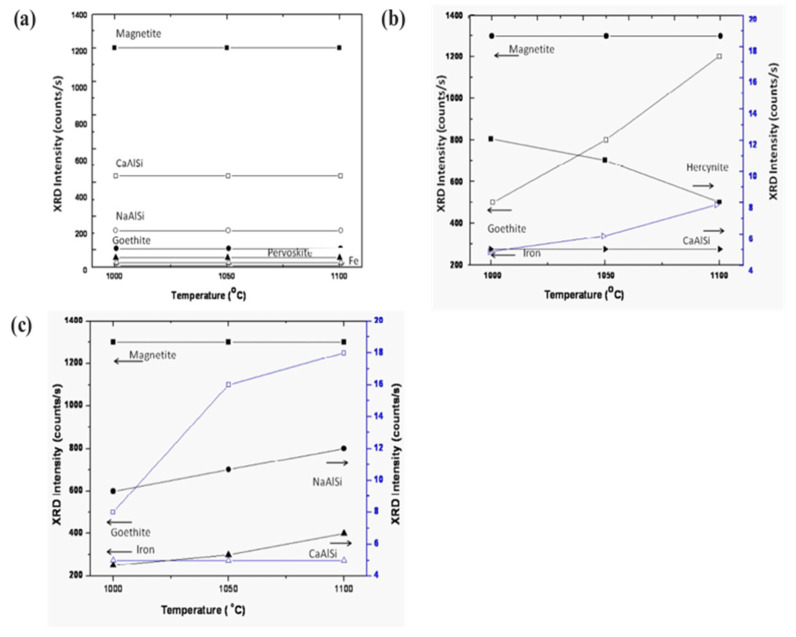
Evolution of various compounds as the function of sintering temperature: (**a**) sintered red mud without any additives; (**b**) sintered red mud with 10 wt.% of fly ash content; (**c**) sintered red mud + 20 wt.% fly ash mixture. Reprint with permission from ref. [55]. Copyright 2015, Elsevier.

Figure 9 displays the various phases of magnetite, calcium aluminum silicate, sodium aluminum silicate, goethite, iron, and perovskite as a function of sintering temperature. The phases show a trending behavior with fly ash mixtures of 10 wt.% (Figure 9a,b). On increasing the percentage of fly ash content (up to 20 wt.%) phase evolution is more stable and distinct (Figure 9c). Metal ions into the various phases are more prominent at lower sintering temperatures without the addition of fly ash [57,58,59]. However, the phases are more distinct and accurate with more specific phases of simpler compounds at a higher sintering temperature of 1100 °C with 20 wt.% of fly ash content. The reduced sintered slag contains various metal ions and mineral sources for the recovery of metal and ions for further utilization in industry [60,61,62].

### 3.6. Carbo-Thermal Smelting Technology

Carbo-thermal reduction of bauxite is developing as a promising alternative technology for the aluminum and aluminum alloy industries. In this process, carbon or coke are used as a reductant for solid-state reduction technology. As a result, metallic iron, ferroalloy of silicon and aluminum, titanium carbides could be obtained as the by-products [19,20]. Based on the smelting technology, we performed previous work on using fly ash additive with the red mud that undergoes sintering technology for the building materials. A combination of smelting and reduction processes allows a reduction in the temperature of 1200–1500 °C to produce slag phase and cast iron if the C content in the cast iron is within the range of 2–2.3%. Another direct route for separation of iron from red mud is the roasting method followed by magnetic separation. Iron (Fe) could be separated from red mud using various methods, either by leaching or by sintering or roasting. As one of the major elements, Fe should be extracted from red mud following various reaction, as outlined below [63,64,65].

Fe_2_O_3_ (s) + C (s) → Fe_3_O_4_ (s) + CO (g) (11)

Fe_2_O_3_ (s) + 3C (s) → Fe (s) + 3CO (g) (12)

CO_2_ (g) + C (s) → 2CO (g) (13)

3Fe_2_O_3_ (s) + CO (g) → 2Fe_3_O_4_ (s) + CO_2_ (g) (14)

Fe_3_O_4_ (s) + CO (g) → 3FeO (s) + CO_2_ (g) (15)

FeO (s) + CO (g) → Fe (s) + CO_2_ (g) (16)

FeO (s) + C(s) → Fe (s) + CO (g)(17)

**Figure 10:**
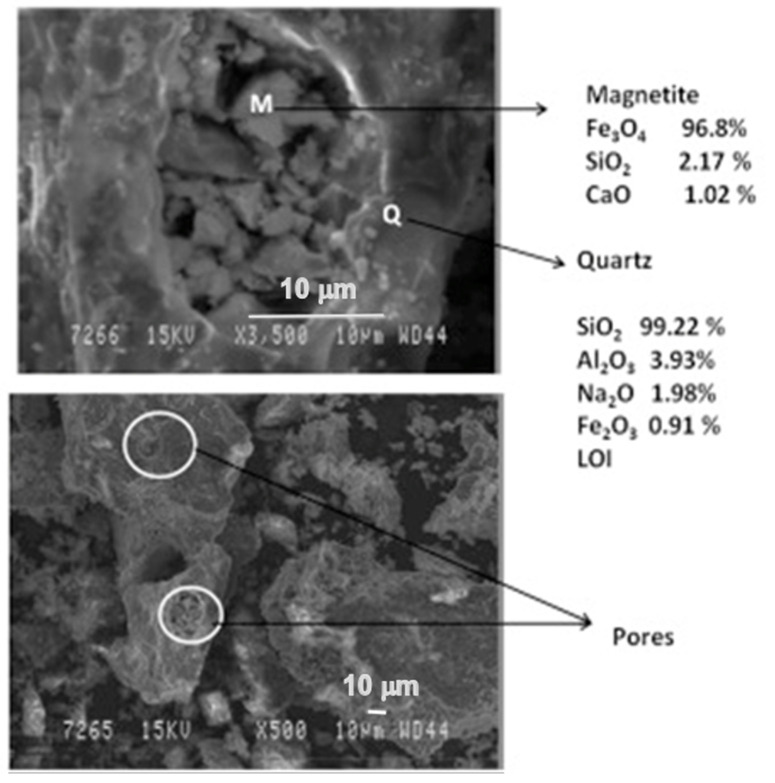
Micrograph of the sintered red mud with 20 wt.% of fly ash content in 1100 °C sintered temperature. Reprint with permission from ref. [55]. Copyright 2015, Elsevier.

The phases were more prominent in the reduced sample of red mud when 20 wt.% of fly ash was used, which is shown in the SEM images reported in Figure 10.

**Figure 11:**
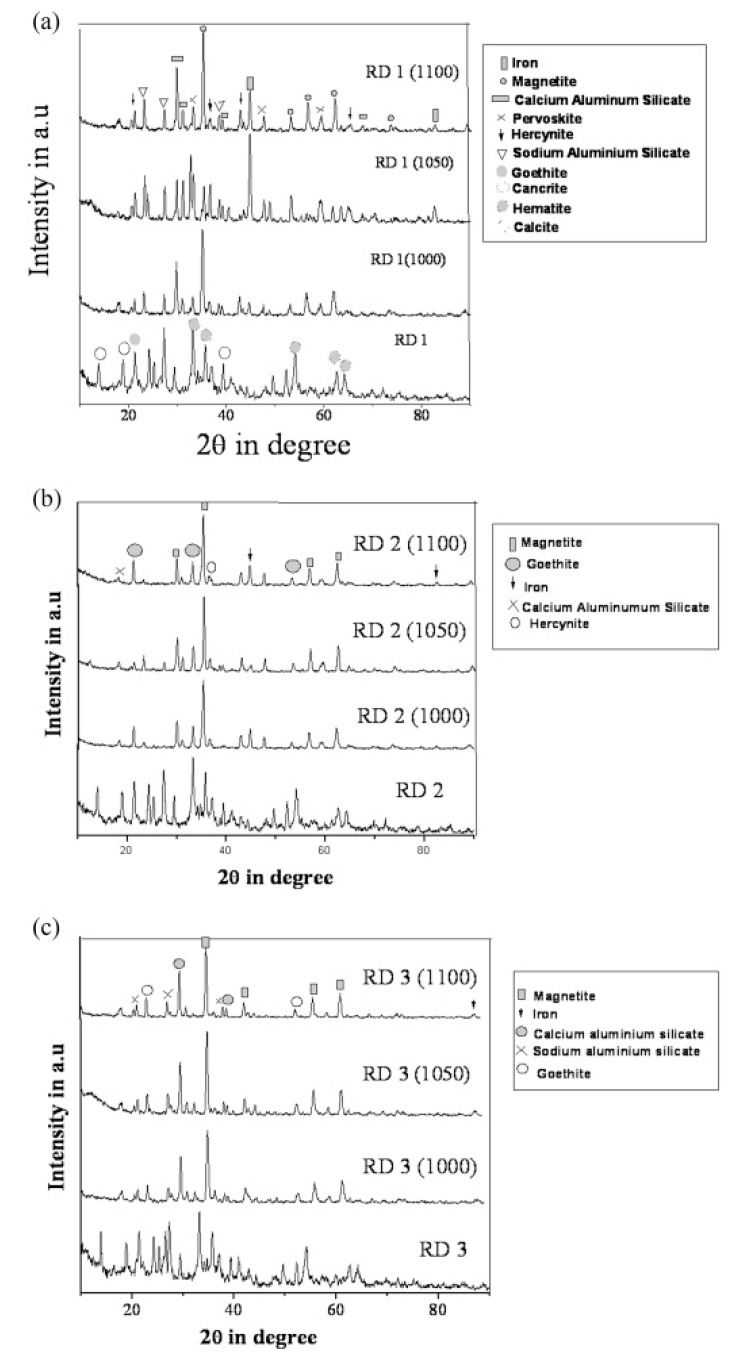
(**a**) Diffraction profile of red mud and sintered red mud at various temperatures; (**b**) diffraction profile of red mud and sintered red mud with 10 wt.% fly ash; and (**c**) diffraction profile of red mud and sintered red mud with 20 wt.% of fly ash content. Reprint with permission from ref. [55]. Copyright 2015, Elsevier.

The evolution of the different phases developed in the reduced sample sintered up to 1100 °C was recorded with the XRD technique and is shown in Figure 11.

Iron is the major element in red mud that could directly reduce the carbon-bearing pellets of red mud with coal at a temperature of 1400 °C for 30 min [66]. The obtained products contain 96.52% iron with low Mn and Si contents. However, P and S contents are high.

## 4. Fields of Application of Red Mud

A considerable environmental concern associated with red mud is associated with its high pH value and its small amounts of heavy metals. These days, the aluminum industries are more focused on producing a cleaner residue from bauxite.

### 4.1. Thermal Plasma Technology for the Production of By-Products from Red Mud

The thermal plasma technology boom, as a prospective area of waste management, is widely reported in the literature and could be used as a technology to reduce the amount of red mud to produce pig iron [67,68,69]. Red mud mixed with carbon graphite undergoes a smelting process to produce pig iron with 71% recovery. This process allows for reduced energy consumption with recovery of metals from the red mud. Waste treatment is considered one of the efficient methods in the energy sector and thermal power plants. Simultaneously, pig iron could be extracted from red mud by adding a fluxing agent of graphite and fluxes [70,71,72].

### 4.2. Mixing Technology for Use of Red Mud as an Additive for Construction Materials

Mixing technology is a methodological way of approaching red mud as an alternative replacement of cement. Partially, red mud can be used in slag for the cementitious material that can be used effectively in building sectors [73,74,75]. Red mud is also considered to add a neutralization effect of the hydration properties of cement materials in the construction industry. Bricks and prisms are some of the resources that could be derived from the red-mud-based geopolymer matrix in areas of the building sector. Red mud is considered as environmentally friendly, self-sensing concrete blended with by-product waste. Red mud is also considered as one of the potential additives for durability and mechanical performance of cement mortars. Geopolymerization of red mud and the slag from ferronickel could emerge as advanced inorganic polymeric material with exceptional physical and chemical properties [76,77].

### 4.3. Separation and Extraction Technology

The separation of the magnetic materials from red mud is considered to be the preliminary step for the separation of iron particles. Simultaneously, extraction technology is more effective in leaching such as chemical technology for the separation of various minerals. The leaching of red mud or sintered red mud is very effective in various sectors for the adsorbents and catalyst categories [78,79,80]. The red mud–fly ash mixtures could be considered as a sustainable acid mine drainage management system. Slag and cement mortar containing non thermally treated dried red mud is considered as opening demand for potential utilization. Red mud is considered an effective additive in geopolymer materials for the adsorption of heavy metal ions.

### 4.4. Coating Technology

Coating technology opens the door for applications in pigment areas using Ti as pigment ion. Red mud could be used as a coating by thermal plasma spray technologies for wear resistance coating or corrosive resistance coating layers that could stand as a barrier against environmental conditions [81,82,83]. Coatings based on red mud offer an important erosion wear resistance, which can further be improved. Red mud could act as deposition material for surface modification technologies in the plating, diffusion process, surface hardening, and thin-film coating sectors. The red mud–polyester composite coating could act as neutron shielding materials from injurious effects of radiation [84,85,86], thus representing an innovative application of red mud in the industrial sector.

Red mud has emerged as a significant contribution to the hybrid composite. Red mud can be used as a resource for transferring a waste-management approach with natural fibers. A hybrid composite concept has been developed for the potential in a red-mud-based geopolymer matrix with the incorporation of fibers [87].

### 4.5. Economic and Social Impact

The potential application of red mud in the various industrial sectors proves that valuable resources will have a significant impact on the economic prospects, boosting economic growth through its potential as a valuable by-product. The social impact will increase only by using it as a resource rather than dumping as waste that holds a threat for environmental pollution [88,89,90]. The significant use of red mud as a resource will reduce the risk of environmental hazards and socially benefit environmental conditions.

### 4.6. Value Recovery and Strategic Utilization

The valuable elements in red mud can be recovered by acid leaching, solid-state carbo-thermic reduction, magnetic and fluidized bed separation, as well as smelting in a blast furnace. In the framework of considering red mud as a resource, we need to improve various steps of metal recovery as one of the potential applications for the pigment industries.

**Figure 12:**
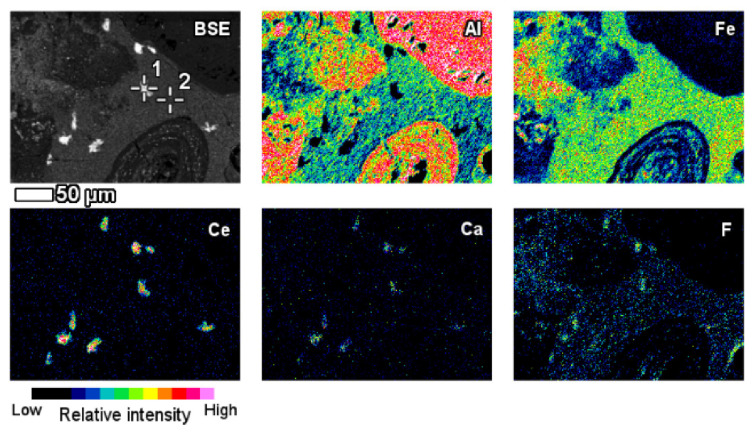
Backscattered image of red mud and elemental image analysis for various elements of Al, Fe, Ce, Ca, F. Reprinted from ref. [91].

The elemental composition and its derivation from red mud, bauxite residue, and their respective Raman spectra for various elements are shown in Figure 12 [91]. A red mud image scan analysis shows minerals and ions present in the sample (Figure 12) [92].

An insight investigation was carried out on various phases of rare earth elements in red mud. This approach leads to a mineralogical insight view with output to improve the rare earth elements recovery process. The distinct rare earth element (REE) phases are also contained within the lateritic bauxite (Figure 12).

**Figure 13:**
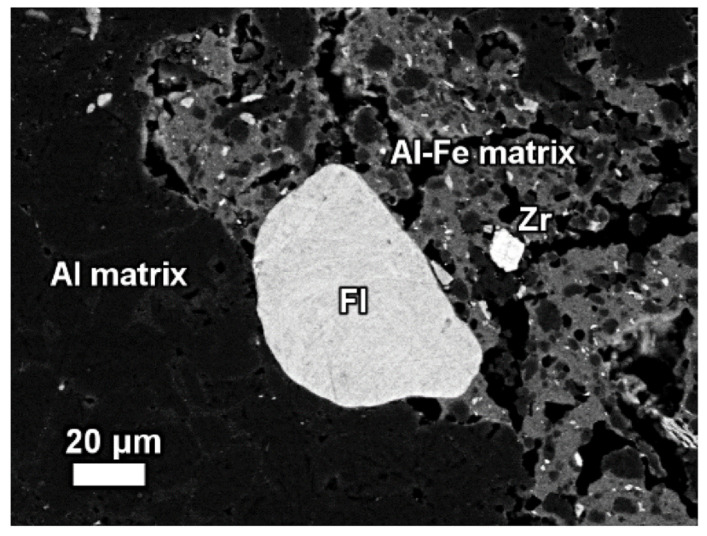
The mineralogical composition of red mud shows the presence of the florencite group of light rare earth elements, grain Zr grain, Al, matrix, and Al–Fe phase. Reprinted from ref. [91].

The REE mineral content includes aluminum, cerium, phosphorous, and then other REEs. Thus, REE phases can be identified as belonging to the florencite group. The compositional analysis of elements is done by EDS spectrum exhibiting a pronounced phosphorus X-ray peak of Figure 13.

The composition of grains resembles rhabdophane–Ce which has been detected in the bauxite phase [93]. REE phosphate do not dissolve easily in sodium hydroxide which is generally used in the metal recovery process of bauxite.

**Figure 14:**
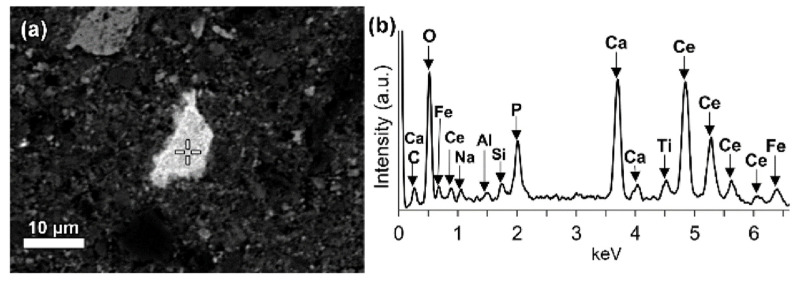
(**a**) Elemental composition of red mud shows the enlarged area of cerium phosphate and (**b**) its respective EDS spectrum. Reprinted from ref. [92].

LREEs are found as calcium containing phosphate phases in bauxite residue, more specifically as cerium phosphates (Figure 14a). It can be seen from the EDS spectrum of an analysed particle, exhibiting a pronounced phosphorus X-ray peak. A wide variation in chemical composition in morphological features is shown in Figure 14b.

**Figure 15:**
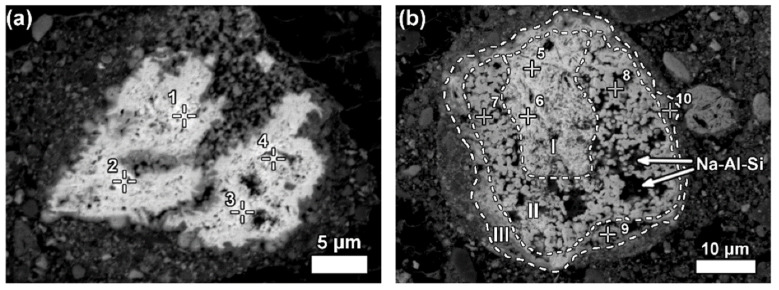
Neodymium–lanthanum as predominant LREE particles, of which (**a**) is partly reacted; and (**b**) exhibits a zonation (I–III) relating to reaction stages with Bayer liquor. Within zone II of (**b**), deposition of a sodium aluminosilicate phase (Na-Al-Si) is indicated. Reprinted from ref. [93].

Some LREE particles contain minor percentages of iron, titanium, and sodium oxide content (Figure 15a). The texture of ferrotitanate grains appears anhedral. Others showed distinct zonation expressed in wide variation in chemical composition as well as in morphological features. Most aggregates of anhedral globular crystallites can be observed on examining larger particles that exhibit a different reaction stage that has been observed in Figure 15b.

**Figure 16:**
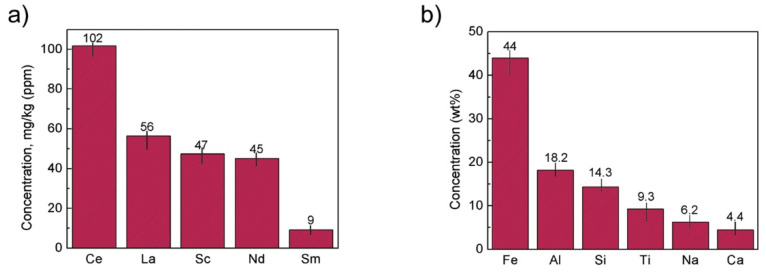
(**a**) REE concentration; (**b**) major element concentration from ICP-OES and XRD elemental composition obtained from red mud. Reprinted from ref. [94].

The concentration of REE elements and major elements from red mud was investigated using ICP-OES and XRD elemental composition (Figure 16).

**Figure 17:**
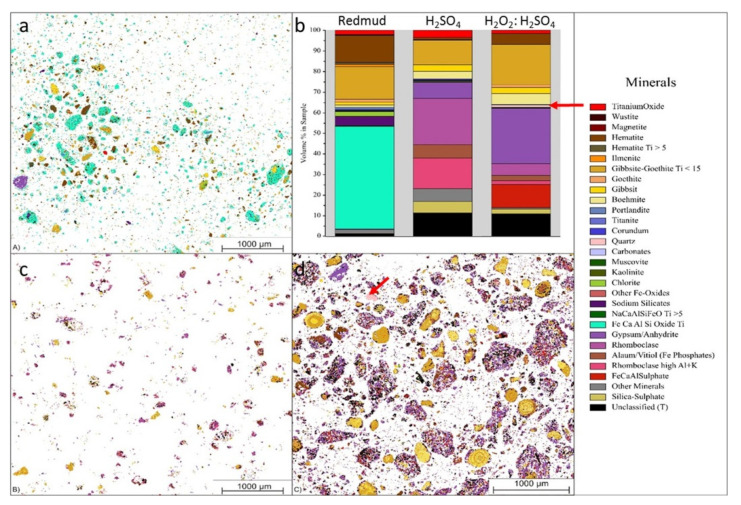
Visual representation of (**a**) the mineralogical distribution of BR; (**b**) phase distribution before and after leaching; (**c**) mineralogical distribution of the leach residue after leaching with 2.5 M H_2_SO_4_; and (**d**) mineralogical distribution of the leach residue after leaching with 2.5 M H_2_SO_4_:2.5 M H_2_O_2_ with S/L = 1/10 at 75 °C for 2 h. Reprinted from ref. [95].

Lightweight alloys for the transportation industry are in serious demand due to their unique and desired properties as alloys. Bauxite residue is considered as a source hub for these alloys with metals with considerable Ti and Sc contents. The combination of hydrogen peroxide (H_2_O_2_) and sulfuric acid (H_2_SO_4_) is used for leaching solution at 90 °C for 30 min to extract Sc and Ti of 68% and 91%, respectively (Figure 17).

Figure 17 displays the three different minerals (red mud, red mud + H_2_SO_4_, Red mud + H_2_O_2_:H_2_SO_4_) that revealed a very distinct distribution within three samples. Red mud shows the presence of Fe, Ca, Al, and Si oxide with high non-stoichiometric intergrowth oxides. When H_2_SO_4_ is incorporated, only Si mineral is detected in addition to others, with almost all Fe in leach residue extract as rhomoclase phase [95,96,97]. On incorporating H_2_O_2_:H_2_SO_4_ leaching solution, the quartz phase is mostly affected with increasing leaching efficiency. There are inhomogeneous particles with particle sizes ranging from 1 µm to 40 µm. Most of the rare earth particles are based on the Fe-based compositions in combination with Ca, Na, Ti (Fe) O compounds [98,99]. However, the minor elements are based on the C, P, Mn also present in the red mud resources Figure 17a,b. Figure 17b represents aggregates of the globular region within the red mud particles that may be caused by a cluster of rare earth elements in the reactive combined stage.

**Figure 18:**
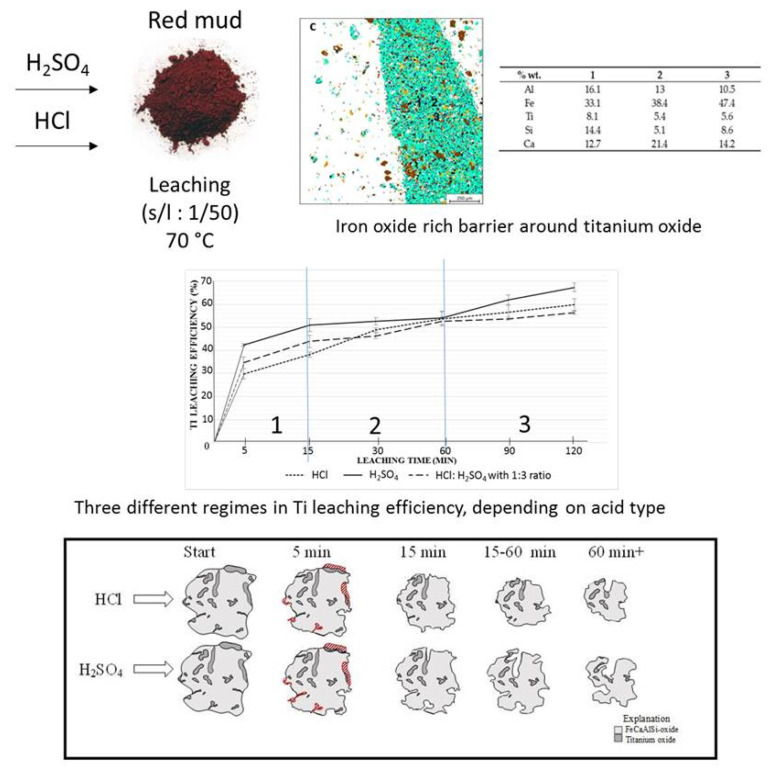
Mineral distribution of red mud shows the presence of various elements and the leaching behavior at various leaching solution and the reacting particle with HCL and H_2_SO_4_ solution as the function of time. Reprinted from ref. [100].

The leaching process is one of the effective ways to extract Ti and Fe from the mineralogical sample. The leaching solution of the sulfuric acid and hydrochloric acid is commonly approved for the extraction process [100]. The 67% extraction of Ti from red mud with H_2_SO_4_ could be achieved by the leaching process [101]. Ti and Fe have different reaction processes in the leaching mechanism for the extraction within the solvent of H_2_SO_4_ and HCl at different rates. The mechanism of the process is represented in Figure 18.

## 5. Discussion

Red mud contains various sources of elements in the category of major, minor, and rare earth elements. In the past 11 years, researchers have been more motivated towards the valuable recovery of metal ions from red mud as resources. Although the primary use of red mud is based on the areas of construction sectors [4,5,16,17,21,22,23,24,43,45,73,74,75], the most valuable secondary concern still arises in the field of metal and mineral sectors. Researchers have focused on various metals present in major and minor quantities in red mud and of which significant amounts could be removed using various processes, such as sintering and carbothermal smelting processes using metallurgical routes [25,26,46,47,48,55,56,57,58,59,60,61,62,63,64,65]. Additional methods, such as the chemical process of leaching, are also investigated as one of the beneficiary ways to extract various categories of elements from red mud [9,22,36,81,90,94,99,100,101,102]. The economic cost of red mud handling and use is one of the important issues associated with the bauxite industry. The general costs of properly handling red mud in some countries are approximately 12 EUR/ton. The general costs of properly handling red mud in various countries are outlined in Table 3.

The amount of funds will increase by government and industry based on the capacity of alumina production. Nevertheless, it is highly beneficial to construct in-house facility for metal extraction that will reduce the expense of transportation of red mud to other location.

## 6. Conclusions

A unique cost-effective and environmentally sustainable technique is very challenging to achieve. The recovery of metals and minerals from red mud using multiple different potential techniques need to be emphasized and implemented. Recovery of Fe (44 Wt.%), Al (18.2 Wt.%), Si (14.3 Wt.%), Ti (9.3 Wt.%), Na (6.2 Wt.%), Ca (4.4 Wt.%) as major elements and of Mg, V, Mn, Cr, K as minor elements, and rare earth elements such as Ce (102 mg/kg), La (56 mg/kg), Sc (47 mg/kg), Nd (45 mg/kg), Sm (9 mg/kg) need to be processed from red mud by use of several steps. The establishment of the red mud industry as potential resources on the recovery of metal ions will open up new strategies for the metal industries and pigment sectors. Although a lot of studies have been carried out on the recovery of metals, this step is still very limited to the laboratory scale. The lab-scale approach needs to be enhanced in commercial ways to recover metals from red mud as resources. The development of neutralization of red mud and the extraction of metals from red mud do require a good understanding of chemistry and the reduction process of red mud.

## Figures and Tables

**Table 1:** Quantification of major elements (wt.%) and minor elements (Conc. Mg/kg) of red mud.

Red Mud Compositions				
Major Elements	Wt.%	Minor Elements	Concentration (mg/kg)	Refs.
Fe_2_O_3_	30–60	U	50–60	[31]
Al_2_O_3_	10–20	Ga	60–80	[32,33]
SiO_2_	3–50	V	730	[32,33,34,35]
Na_2_O	2–10	Zr	1230	[36,37]
CaO	2–8	Sc	60–120	[38]
TiO_2_	8.50	Cr	497	[38]
P_2_O_5_	0.25	Mn	85	[39]
MgO	0.10	Y	60–150	[39,40]
K_2_O	0.06	Ni	31	[38]
–	–	Zn	20	[38]
–	–	La	0.1–1%	[41]
–	–	Th	20–30	[42]

**Table 2:** Major elemental composition of red mud from various locations in the countries.

Composition wt.%														
Location	Al_2_O_3_	Fe_2_O_3_	SiO_2_	TiO_2_	CaO	Na_2_O	Mn	P_2_O_5_	V_2_O_5_	Gd_2_O_3_	MgO	K_2_O	LOI	REFs
Ajka Aluminum Industry, Hungary	16–18	33–48	9–15	4–6	0.5–3.5	8–12	-	-	0.2–0.3	-	0.3–1	-	-	[35]
Aluminium Pechiney, Gardanne, France	15.00	26.62	4.98	15.76	22.21	1.02	-	--	-	-	0.95	-	12.10	[37]
Bauxite ore refinery, Guinea	26.60	48.40	5.50	-	1.30	-	-	-	-	-	0.90	-	14.60	[36]
ALCOA factory, San Cibrao (Northwest of Spain)	12.00	37.00	9.00	20.00	6.00	5.00	-	-	-	-	-	-	-	[39]
Korea Chemical Co.	23.70	16.60	22.90	6.70	6.70	11.60	-	-	-	-	-	-	-	[40]
Shandong Aluminium Factory, China	7.96	6.57	21.90	-	38.84	2.32	-	-	-	-	1.60	0.41	17.42	[41]
Greek red mud, Greece	15.60	42.50	9.20	5.90	19.70	2.40	-	-	-	-	-	-	-	[42]
Slurry pond from Worsley Alumina, Australia	15.00	60.00	5.00	5.00	-	16.00	-	-	-	-	-	-	-	[43]
Alpart factory and the Alcan Ewartonred mud pond, Jamaica	18.80	45.30	4.30	6.40	3.10	1.50	-	-	-	-	-	-		[44]
Shandong Aluminium Corporation, Shandong, China	6.93	12.76	19.14	3.43	46.02	2.37	-	-	-	-	1.15	1.20	5.73	[45]
Alumina-aluminio of San Ciprian, Lugo, Spain	20.10	31.80	6.10	22.60	4.78	4.70	-	-	-	-	0.20	0.03		[46]
Etibank Seydiehir Aluminium Plant, Konya, Turkey	20.39	36.94	15.74	4.98	2.23	10.10	-	-	0.05	-	-		8.19	[47]
Aluminium of Greece S.A.	15.65	45.58	6.96	7.07	14.84	3.26	-	-	-	-	-	0.07	-	[48]
Eurallumina alumina plant, Italy	17.19	30.45	9.58	8.61	7.77	12.06	-	-	-	-	0.86	0.30	12.38	[49]
Queensland Alumina Ltd. refinery, Gladstone, Australia	25.45	34.05	17.06	4.90	3.69	2.74	-	-	-	-	1.86	0.20	-	[50]
Seydiehir Aluminium Plant, Konya, Turkey	14.10	38.30	2.50	-	4.10	-	-	-	-	-	-	-		[51]
HINDALCO Renukoot, India	21.9	28.1	7.5	15.6	10.2	4.5	–	–	–	–	-	-	12.2	[52]
IND ALMuri, India	24.3	24.5	6.2	18.0	–	5.3	–	–	–	–	-	-	–	[52]
BALCO Kobra, India	19.4	27.9	7.3	16.4	11.8	3.3	–	–	–	–	-	-	12.6	[52]
NALCo Damanjodi, India	14.8	54.8	6.4	3.7	2.5	4.8	1.1	0.67	0.38	0.01	-	-	9.5	[52]
INDALBelgam, India	19.2	44.5	7.0	13.5	0.8	4.0	–	–	–	–	--	-	10.0	[52]
MALCO Mettur Dam, India	14.0	18.0	56.0	50.0	2.0–4.0	6.0–9.0	–	1.0–2.0	–	–	-	-	12.60	[52]

**Table 3:** General cost of properly handing RM in some countries. Reprinted from ref. [103].

Country	Annual Output of Red Mud (Million Tons)	Cost (Million EUR)/Year
Australia	30	373
India	10	125
Brazil	10.6	132
China	88	1100
Greece	0.7	8.72

## Data Availability

Not applicable.
